# Elements of Pregnancy and Parenthood Policies of Importance to Medical Students and Included in a Sample of Medical Schools' Websites and Student Handbooks

**DOI:** 10.1089/whr.2021.0105

**Published:** 2021-11-29

**Authors:** Jessica L.R. De Haan, Franklin Dexter, Bradley M. Fleming, Amy C.S. Pearson, Suzanne D. Reuter

**Affiliations:** ^1^ Department of Anesthesia, University of Iowa Carver College of Medicine, Iowa City, Iowa, USA.; ^2^ Department of Anesthesia and Health Management and Policy and University of Iowa, Iowa City, Iowa, USA.; ^3^ Department of Department of Anesthesia, University of Iowa, Iowa City, Iowa, USA.; ^4^ Department of Pediatrics, University of South Dakota Sanford School of Medicine, Sioux Falls, South Dakota, USA.

**Keywords:** pregnancy, parental leave, human resources, quality assurance

## Abstract

***Background:*** Medical students who are parents or considering parenthood often want information about school policies. An earlier survey of 194 medical students from one U.S. school examined seven “elements that [students thought] should be included in a school policy on pregnancy/maternity leave.” For example, students want to know “how much time a student can take off during medical school and still graduate with their class.” We performed multivariate and multivariable analyses of the University of South Dakota survey to understand its generalizability and usefulness.

***Methods:*** The earlier survey also included 35 demographic variables about individual students. We tested empirically for associations between the demographics and the seven policy items, thereby evaluating generalizability of the survey results to different demographic groups. We then surveyed public websites of a sample of U.S. medical schools to evaluate usefulness of the knowledge of the seven items. For the 33 surveyed schools, we documented if each of the items was present on publicly available webpages and handbooks.

***Results:*** The seven items had content validity as a necessary and sufficient set of items. There also were no significant associations of the items with demographic variables. Therefore, there is little chance that differences among medical schools in their average demographic would affect the items needed for their websites and student handbooks.

Among the surveyed medical school websites, 1 of 33 had all seven items (upper 95% confidence limit: 14% of schools nationally would be expected to have all seven items shown).

***Conclusions:*** These findings show that it is known what information students want to know about in a school policy on pregnancy and parental leave. Adding these items to public websites is a necessary and an easily actionable intervention to help current and future medical students.

## Introduction

More than 1000 (≅7%) of U.S. medical students graduating in 2020 were parents.^1^ Nevertheless, most medical school lack publicly accessible policies about parental leave^2^ and associated topics that students need to know when choosing schools and coordinating plans for parenthood and schooling. The purpose of our study was to investigate what content to include in medical schools' policies about pregnancy, breastfeeding, and maternity leave. The study addresses a recent needs assessment for support of medical students who are parents and/or planning parenthood.^3^

A survey of 194 medical students at University of South Dakota Sanford School of Medicine in 2017 included seven “elements that [surveyed students thought] should be included in a school policy on pregnancy/maternity leave” (Table 1).^4^ In the current study, we first performed new statistical analyses of the previously published survey data to evaluate if the survey results are generalizable to various demographic groups. We hypothesized lack of association between each item to be included and responses to the other questions in the survey. The basis for our hypothesis was that medical students have sufficient experience to know, regardless of gender or background, what pregnancy and childcare entails (e.g., there is birth followed by lactation).

**Table 1.** Distribution of the seven items among the 33 studied medical schools' websites

Items that “you think are the key elements that should be included in a policy on pregnancy/maternity leave”^4^	Median^a^	75th percentile
The school's plan for communicating the policy to the students^b^	1	2
How a student arranges for maternity/paternity leave	0	2
How the student receives/requests additional accommodations/flexibility if pregnancy or breastfeeding interferes with expected activities^c^	0	2
A statement indicating that the medical school wishes to be supportive of pregnancy and parenthood in medical school^d^	0	1.5
How the student and school jointly devise a plan to make up required elements that are missed during leave or because of pregnancy	0	1
How much time a student can take off during medical school and still graduate with their class^e^	0	1
Resources available for students who desire mental health counseling^f^	0	0

^a^ The items are listed in the table in descending sequence of the sum among the 33 programs on the zero, one, or two scale used in the Adobe Acrobat PDF files accessible at https://doi.org/10.25820/data.006159.

^b^ This item does not specify the scope of the policy. The preamble listed in the first row refers to “pregnancy/maternity leave.” Therefore, when assigning the scores of zero, one, or two for this item, the scope used was for any school policies available on its public website referring to “pregnancy” or “maternity leave.”

^c^ The item referred to “pregnancy or breastfeeding,” and therefore “or” was used when assigning the scores of zero, one, or two for this item. The item refers to “additional accommodations/flexibility” and “interferes,” and therefore a map or statement of location(s) of lactation rooms were not considered as meeting the criterion.

^d^ This item refers to “pregnancy” and “parenthood.” The preamble listed in the first row refers to “pregnancy/maternity leave.” Therefore, when assigning the scores of zero, one, or two for this item, a statement that supports lactation but had no mention of pregnancy, maternity leave, paternity leave, or another category of supporting parents was treated as a score of zero.

^e^ Time off for the purposes of “childbirth” or “adoption” was treated as equivalent wording for “maternity leave” when assigning the scores of zero, one, or two for this item.

^f^ For the last item, a response of one or two (see Methods) was given if the medical school website referenced mental health resources or counseling for pregnant, postpartum, or parenting students, matching the instructions in the originally performed survey: “a policy on pregnancy/maternity leave.”^4^

We then surveyed public websites of medical schools to estimate what percentage of schools included all seven items that students viewed as necessary (Table 1). If schools already included the seven items on their own, the knowledge that there are seven items to be included would be valid but not useful information to guide school actions. We, however, hypothesized that most schools have not relied on and/or known about the knowledge of the seven items and thus the survey results are not obvious. Rather, they are scientifically useful. The basis for our hypothesis was limited citation of the earlier survey study^4,5^ despite calls for precisely this information.^3^ If the hypotheses were supported, adding these items to schools' public websites would be an easily actionable intervention to help current and future students who are parents or are considering parenthood.

## Methods

The University of South Dakota IRB approved the original, previously published study and the new analyses of the previously collected data.^4^ The University of Iowa determined that the new collection of data from publicly available websites does not meet the regulatory definition of human subjects research.

### New analyses of data previously collected by Bye et al.

A copy of the full original survey is at https://doi.org/10.25820/data.006159 and in Supplementary Data. Their survey's item #9 included the seven items “that should be included in a policy on pregnancy/maternity leave” (Table 1).^4^ Their response rate was 194 out of 249 medical students (78%).^4^

The seven items of interest were binary, to be “included” or not. Internal consistency was evaluated using the Kuder–Richardson coefficient of reliability, such as Cronbach's alpha, but for binary data.^6^ Calculations were performed using the STATA function kr20 (StataCorp, College Station, TX).

The seven items were followed by a choice to select Other and enter free text.^4^ These additional themes listed more than once are described, because frequency of a missing theme would limit validity of use of Table 1. This was important because Bye et al. chose the seven items during a brainstorming session by the authors,^4^ including the principal investigator who was pregnant and had a child while she was enrolled in medical school.

The survey also included 70 other response categories to 35 demographic variables and other questions that could plausibly have been associated with the seven items (Table 2).^4^ All 245 combinations were evaluated empirically for statistically significant association using Cramér's V for effect size and Fisher's two-sided exact test for inference. The rationale is that if results were sensitive to medical student demographics, items important to students at some medical schools may be less important to students at other schools. The fact that schools need to address the needs of diverse groups of students highlights that only demographic variables having a large effect on ratings of the importance of the seven items would be relevant.

**Table 2.** The other 35 survey responses listed in same sequence as in the survey and the supplementary data

Variable	Responses
Are you male or female?	194
How many children do you have?	194
Are you planning to have additional children during medical school?	191
How many children did you have before coming to medical school?	192
Are you (or your significant other) currently pregnant?	192
Were you (or your significant other) pregnant when you started medical school?	193
If you have a significant other, is he/she also a medical student?	194
Does your medical school have a written policy/document addressing pregnancy/maternity leave during medical school?	194
Are you a parent or are you (or your significant other) currently pregnant?	194
Do you receive any help from medical school in terms of being a parent?	52
If you currently have children, what do you utilize for routine daily childcare? Significant other?	194
Daycare?	194
Other family member (mother, aunt, etc.)?	194
Friend?	194
Nanny?	194
Preschool/Montessori?	194
Were/are you or significant other pregnant at any time during medical school?	43
	194
Did you receive any support from the medical school in terms of pregnancy or the postpartum period?	27
	194
If you had a complication with your pregnancy or labor and delivery, did your provider require you to take time off?	16
	194
If yes, what was the provider-required length of time?	12
	194
How much time did you take off during the pregnancy, which was not required by a provider?	17
	194
How much time did you take off for maternity/paternity leave after delivery?	18
In retrospect, would you have taken more time off for maternity or paternity leave if additional time off did not delay graduation?	194
… if additional time off reduced the number of elective rotations you could take but did not delay graduation?	194
… if additional time off delayed graduation by 1 month?	194
Has pregnancy/parenthood affected your decisions with regard to specialty choice?	194
Has pregnancy/parenthood affected your decisions with regard to choice of electives?	194
Has pregnancy/parenthood affected your decisions with regard to choice of away rotations?	194
What insurance produce did/do you use for you or your significant other's pregnancy?	24
	194

### Systematic review of public medical school websites

Initially, the public websites of the authors' (J.L.R.D.H./B.M.F.) home institution and several other large U.S. state schools were reviewed. Using those observations for statistical power analysis, we hypothesized that, as observed, none (0) of the schools would include the full set of seven items desired by students in the previously published study.^4^ If none (0) of *N* = 33 schools' websites mention all seven items, then the upper 95% one-sided confidence limit using Clopper-Pearson confidence interval would be <10%.^7^ Because we were considering observations of literally 0 successes, the Clopper-Pearson upper limit is an exact method.^7^ Therefore, we recorded the features of *N* = 33 medical schools' websites.

The population used was the U.S. News & World Report 2021 Best Medical Schools: Primary Care ranking.^8^ This strategy of selecting a population of schools for study from U.S. News & World Report matches that used by several recent research letters in JAMA, recording childbearing and family leave policies for resident physicians, employees, and administrative staff of medical schools, although none used a systematic, studied set of criteria.^9–11^ (See Limitations for more Discussion).

There were three schools tied for 31st to 33rd, but only one school ranked 34th. Thus, there conveniently was a precise set of schools that were the “top” 33. The University of South Dakota was not included among these *N* = 33 schools; therefore, there was not potential for bias due to overlap of the survey population and the applied population. We have included an Excel file with details at https://doi.org/10.25820/data.006159.

Each of the medical school's website pages were screened for the seven items by entering one of the following sets of text into the Google search box. One Google search protocol was:

(“student handbook” OR (policies procedures)) (pregnancy OR breastfeeding OR “maternity leave” OR “parental leave”) site:[medical school domain]

As used below for scoring, this search identified items directly. The other search protocol relied on subsequent navigation through multiple links on schools' websites:

(“student handbook” OR (policies procedures)) site:[medical school domain]

For these Google searches, “AND” is inferred. Specification of the site (e.g., “site:medicine.uiowa.edu” for University of Iowa) limited the search to the medical school's domain. Bing uses the same commands. Adobe Acrobat PDF files showing the Google Search were generated and kept for posterity, as were web pages addressing the seven items. Searches were done over a couple of months, without being logged into any specific Google user account.

Medical school web sites change over time. Therefore, all PDF files were checked on February 13, 2021, for any changes to the websites, and updated.* Repeating on a single date yielded a single, up-to-date, cross sectional survey of the 33 medical schools' public websites on that one date. These PDF files (i.e., our results) are available for readers at https://doi.org/10.25820/data.006159. They include the web searches, PDF printouts of the schools' website pages, and downloaded PDF policy manual(s). Policies applied were those for medical students *per se*, not when listed solely about medical students in federally funded training programs (e.g., Medical Scientist Training Program).

Presence of the seven items desired by students were highlighted in each school's PDF file using Adobe Acrobat PDF highlight tool.^4^ No medical schools were contacted for the study; the information collected was limited to the medical schools' public (internet) websites. Each of these PDF files was reviewed by both J.L.R.D.H. and B.M.F. The scoring is available in the Microsoft Excel workbook at https://doi.org/10.25820/data.006159, and in the corresponding highlight comments in the PDF files.

For each of the seven items, the counts reported are zero, one, or two. The score of two was entered when the item was identified directly by the first search criteria, above. A score of one was assigned when the item was found by the second search criteria (i.e., navigating through multiple links on school websites/handbook). A score of zero was assigned when the school does not have a public entry for the item. Thus, each of the *N* = 33 schools' websites were summarized by seven scores, each score being zero, one, or two (see Results, next paragraph). Statistical analyses using Clopper-Pearson method (above) were based on the counts of items with score of either one or two.

## Results

### More information about the seven items in University of South Dakota survey^4^

The estimated Kuder-Richardson coefficient of reliability was 0.74 (*N* = 194 respondents among the 249 medical students).^6^ Table 1 was sorted in sequence of the observed mean scores among the 33 studied medical schools. “The school's plan for communicating the policy to the students” in the top row of Table 1 had the smallest observed correlation with the sum of the rest of the items' scores, 0.35. “Resources available for students who desire mental health counseling” in the bottom row had the median observed correlation with the rest of the items, 0.46. These observations show both that the seven items are not homogeneous and no one of the seven items should be treated as more important or less important than the others.

There were 6 of 194 respondents who listed another theme. Three of these were singletons. Two respondents requested information about local daycare options, and a third requested financial planning recommendations related to childcare (i.e., a similar concept). The fact that 3/194 is much less than the counts (127 to 184) among the 194 respondents for the seven included items suggests content validity of the seven items.

### Evaluation of generalizability

The 245 associations between the seven items in Table 1 and the 35 potential demographic covariates were calculated. The statistical output is provided in a PDF file at https://doi.org/10.25820/data.006159, in the same sequence as in Table 2. The 16 associations that had Fisher's exact test *p* < 0.05 are listed in Table 3. None was statistically significant with adjustment for the false discovery rate. Therefore, the new analyses of the previously collected survey data suggest generalizability of the findings to medical schools with different demographics for their average student.

**Table 3.** The 8 of 245 combinations of the seven items and 35 other survey responses with unadjusted *p*-values <0.05^a^

Item from among the 7 items in Table 1	Other variable from among the 35 such variables in the survey^b^	Responses by the other variable, % Yes for the item, and counts	Cramér's V^c^	*p*-Value^b^ false discovery rate adjusted	*p*-Value^b^ familywise error rate adjusted
How the student and school jointly devise a plan to make up required elements that are missed during leave or because of pregnancy	Were/are you or your significant other pregnant at any time during medical school?	Yes, 100% (25/25) No, 67% (12/18)	0.47	0.62^c^	0.62
How the student receives/requests additional accommodations/flexibility if pregnancy or breastfeeding interferes with expected activities	If you have a significant other, is he/she also a medical student?	Yes, 95% (20/21) No, 90% (112/125) No significant other, 71% (34/48)	0.25	0.45	0.90
How the student receives/requests additional accommodations/flexibility if pregnancy or breastfeeding interferes with expected activities	Are you male, female, or other?	Male, 79% (83/105) Female, 93% (83/89)	0.20	0.47	0.99
The school's plan for communicating the policy to the students	Are you planning to have additional children during medical school?	Yes, 96% (26/27) No, 77% (126/164)	0.17	0.96^c^	0.99
How a student arranges for maternity/paternity leave	Are you male, female, or other?	Male, 91% (96/105) Female, 99% (88/89)	0.17	0.89	0.99
The school's plan for communicating the policy to the students	Were/are you or your significant other pregnant at any time during medical school?	Yes, 96% (24/25) No or Unanswered, 78% (131/169)	0.15	0.99	0.99
How much time a student can take off during medical school and still graduate with their class	Are you male, female, or other?	Male, 88% (92/105) Female, 97% (86/89)	0.16	0.99	0.99
A statement indicating that the medical school wishes to be supportive of pregnancy and parenthood in medical school	Are you a parent or are you (or your significant other) currently pregnant? If Yes, how much time did you take off for maternity/paternity leave after delivery?	<1 week, 100% (13/13) 1–4 weeks, 0% (0/1) 5–6 weeks, 100% (2/2), >6 weeks, 50% (1/2)	0.85	0.99	0.99

^a^ The 245 combinations were each tested for association using Fisher's exact test. The eight items in this table are those with the unadjusted *p* < 0.05. The STATA 16.1 results are provided at: https://doi.org/10.25820/data.006159

^b^ There were seven items × 35 demographic variables = 245 combinations. The count of 35 demographic variables was from 29 questions plus six variables that were intentionally missing because neither the respondent nor partner was pregnant during the respondent's time so far in medical school. The six extra variables were filled in and treated as separate for inference. The items in this table are those with unadjusted, univariate *p* < 0.05.^a^ For purposes of adjusting the false discovery rate using the Benjamini-Hochberg procedure and adjusting the familywise error rate using the Hochberg step-up procedure, the number of comparisons was treated as 203, where 203 = 7 × 29.

^c^ The table is sorted in ascending sequence of the unadjusted *p*-values, 0.0030 to 0.039. The adjusted *p*-values are not monotonic by our use of the Benjamini-Hochberg stepwise procedure.

^d^ Cramér's V is a measure of association between categorial variables, between 0 and 1. The largest observed value has the largest unadjusted *p*-value, ^c^ because of the small sample sizes, shown in the third column.

### Evaluation of methodologic validity of web site survey

Kraus et al. surveyed medical schools' websites in 1999 and found using different methods that 33% of medical schools (65/199) had parental leave policies listed on their websites.^2^ We similarly found, for the two related of the seven items, that 45% (15/33) had information about “how a student arranges for maternity/paternity leave” and 27% (9/33) had “how much time a student can take off during medical school and still graduate with their class” (Table 1).

### Evaluation of usefulness of knowledge of the seven items

Among the studied U.S. medical school websites, 1 of 33 had all seven items. Thus, from the one-sided 95% binomial confidence interval, at most, 14% of medical schools nationally would be expected to have all seven items. This finding that many fewer than half of schools (*p* < 0.0001) independently included all seven items of concern shows usefulness of the study of the seven items (Table 1). Furthermore, for six of the seven items, the median school lacked the item, and for the seventh item the median school did not have it findable by Google search or student handbook.

## Discussion

We performed multivariate and multivariable analyses of a subset of Bye et al.'s University of South Dakota medical student survey “on pregnancy/maternity leave.”^4^ (The original report in South Dakota Medicine reported raw counts and percentages).^4^ From the reliability analysis, the seven items of Table 1 are a necessary and sufficient set of items. In addition, because individual students' demographics did not significantly affect the findings (Table 3), there is little chance that differences among medical schools in their average student demographics would affect the items needed for their websites and student handbooks. Applying the seven items to a systematic series of medical schools, the vast majority (32/33 observed) failing to include all seven items on their own shows the usefulness of knowing the items of Table 1.

### The seven items and their generalizability

The organization of The University of South Dakota Sanford School of Medicine and the geography of South Dakota was a strength for testing items in pregnancy and parental leave policies. There was lack of association between (1) selecting an item as important knowledge and (2) responses to the other questions. Lack of association supports generalizability of the findings to other schools, provided there was large sample sizes of responses from the studied school (with one set of policies); see Limitations section, below. Bye et al. probably achieved such a large sample size at least partly because the University of South Dakota Sanford School of Medicine has multiple campuses.^12^

As context to readers from the U.S. East Coast, the driving distance from the Rapid City campus and its rotations to those in Vermillion is the same as Baltimore, MD, to Boston, MA. The distances being so long (e.g., vs. those reflecting daily commuting) magnifies the importance of items in Table 1 (e.g., “expected activities” and “required elements” reliably affect how medical students would coordinate obstetrical appointments, spouse/partner residence and job, and daytime childcare). However, there may have been other cultural or institutional values that played a role in the large survey response.

There are added data suggesting the content validity of the seven items in the University of South Dakota survey (Table 1). After the original survey was published,^4^ Sterling and Allan described construction and validation of a multidimensional quality of maternal leave scale.^13^ Among its six dimensions, benefits including financial compensation would not apply to medical students.^13^ The other dimensions (time off, flexibility, coworker support, discrimination, and microaggressions)^13^ highlight the importance of “a statement indicating that the medical school wishes to be supportive of pregnancy and parenthood in medical school”^4^ in Table 1. From Sterling and Allan's dimensions, such a statement should include support for parenthood, assuring that students are not treated negatively, and advising that students and faculty are not shown unnecessary concern about students' personal, family decisions.

There also was a qualitative study of Australian medical students, published after the original University of South Dakota survey.^4,14^ Most of the students with dependents felt that their career would significantly impact their ability to achieve a work-life balance, and that parental leave policies at any career state were inadequately supportive.^14^ The inclusion in the seven items (Table 1) also is consistent with content validity.

### Data about usefulness of knowing the seven items of Table 1

From our study, we expect (*p* < 0.0001) that many fewer than half of U.S. medical schools currently include all seven items important to medical students about pregnancy and parental leave during medical school in publicly available websites or policy handbooks. Schools, despite their likely considerably good intentions, do not reliably include the information. Therefore, we recommend that they take advantage of the University of South Dakota findings^4^ for their information easily searchable by prospective medical students.

There have been earlier systematic but nonrandomized or complete studies of similar characteristics for medical schools.^9–11^ For example, Riano et al. reported paid family leave policies for employees of 12 medical schools.^9^ Magudia et al. recorded childbearing and family leave policies for resident physicians at 15 hospitals offering graduate medical education.^10^ Vance et al. listed paid childbearing and family leave policies for administrative staff.^11^ Our study of medical schools' websites and downloadable materials provided comparable information, but for medical students, and more importantly, for different goals and with a different statistical design.

Our work is closest to the recent study by Kraus et al., wherein 33% of medical schools (65/199) had parental leave policies listed on their websites; our results matched showing validity of our different methodology. Our current study adds to the knowledge of these earlier studies^2,9–11^ by showing what information to provide in listed searchable, public policies on medical student pregnancy and parenthood. Our study was distinct from Kraus et al.^2^ in that they established the presence of any parental leave policy at each school, compared with the content of the policies.

Medical students are adults of heterogeneous life stages and, consequently, information about elements of pregnancy and parenthood policies are needed. Among the four classes of medical students at the University of South Dakota Sanford School of Medicine during the 2016–2017 academic year, there were 249 total students.^4^ Among the 194 respondents (89 of whom were women), there were 29 parents or currently pregnant (or partners pregnant), 12%.^4^ Among 96 of 183 women first-year (2019) medical students at Federal Fluminense University in Rio de Janeiro, Brazil, responding to a survey, two had children, 2%.^5^ Among U.S. medical students graduating in 2020, the percentage with dependents was 7%.^1^ The fact that these percentages represent thousands of students and indicates the value of systematic study of what items to include in pregnancy and parental leave policies.

### Limitations

The instructions for the seven items were to select those that “you think are the key elements that should be included in a policy on pregnancy/maternity leave.” The instruction does not include paternity leave. However, the content analysis did not suggest that a single respondent found this confusing given that items included “How a student arranges for maternity/paternity leave.”

Some of the 35 other survey responses were uncommon. Given the large sample size, this was unimportant. For example, consider the item “How a student arranges for maternity/paternity leave” (Table 1), considered to be a “key element to be included” by 184/194 respondents. If none of the remaining 10/194 had a demographic characteristic versus all of the 184/194, or vice-versa, then even with the adjustment for the 203 characteristics (Table 3), still *p* < 0.0001.

In fact, even if only 3/194 had the demographic characteristic and did not consider the item important, 7/194 lacked the demographic characteristic and did not consider the item important, and 184/194 lacked the demographic characteristic and considered the item important, that uncommon characteristic would still be significant, Fisher's exact test adjusted *p* = 0.020. Therefore, the fact that none of the 35 other responses was significantly associated with choice of the seven key elements to be included shows that our results reasonably can be generalized to other U.S. medical schools.

The population of 33 schools surveyed were those with top ranking in U.S. News & World Report 2021 Best Medical Schools: Primary Care.^8^ U.S. News & World Reports has another ranking for the top medical schools based on research. We picked the primary care ranking, because graduate students and MD/PhD students supported by U.S. federal research or training grants have different (required) family and maternity leave policies. They have different schedules and often fewer geographically dispersed rotations than most medical students. In addition, primary care includes women's health, integral to our topic of interest.^8^ Future studies could use all schools like those done by Kraus et al.^2^

Although we compared our findings to Kraus et al. for purposes of confirming methodological validity,^2^ presence of a parental leave policy was simply a necessary condition for the related, specific items of content desired by students: “how a student arranges for maternity/paternity leave” and “how much time a student can take off during medical school and still graduate with their class.” Assessing for the presence of a policy can be done more quickly.

Thus, the earlier study^2^ searched all U.S. medical schools. In contrast, evaluating the content of those policies and scoring each medical school's website(s) takes hours. However, there was no need to study all schools because our primary goal in reviewing websites was to assess usefulness of the scientific knowledge of what students want to know. Future study could evaluate the prevalence of these seven items among all schools for the different purpose of assessing whether accreditation should encourage that such information be provided.

For assessing usefulness (i.e., prevalence of items), we included not only webpages and PDF files obtained directly by Google search but also by manual clicking (see Methods' footnote *). We therefore include the generated Adobe Acrobat PDF files that are available for review by any reader at https://doi.org/10.25820/data.006159. These are documented with comments, each item checked by two authors (J.L.R.D.H., B.M.F.). We also limited our Results to findings that are likely reproducible, specifically that many fewer than half the schools included all the items.

However, doing so downplayed that a policy unsearchable with logical terms and that neither students nor staff can find lacks sufficient accessibility to be useful, but we counted it nevertheless (Table 1). For example, the school with all seven items was initially overlooked by one study author because the school uses two public websites, but the one with all seven items was blocked from Google search and search within the school's own site. Although this may prevent its usefulness for prospective students, our counting its presence highlights our findings that elements important to students and key personnel may be difficult to find even if present.

We interpreted our result that only 1 of 33 surveyed schools had all seven items listed, and the one listing all items did so with the information challenging to find* as showing that knowledge of the seven items is useful to form policies fitting with the needs of medical students who are parents or considering parenthood. Otherwise, more schools would have had the information public. However, there is an alternative interpretation to our finding. Hypothetically, most schools fully lack concern or consideration that their students are adults, many contemplating becoming or already being parents. The fact that this alternative explanation seems unlikely, notwithstanding being condescending and inflammatory, highlights the validity of our interpretation of incidence as showing usefulness of the knowledge of what items students want to know.

Finally, future study could evaluate whether medical schools supplying all desired information publicly results in more students choosing to have children earlier, thereby reducing the risk of infertility. However, the survey may lack the data needed to evaluate the policy for that purpose because age was not one of the included demographic variables. Survey questions had to ask each respondent their sex, whether a current parent, whether recently pregnant, family plans, significant other, and if also a medical student. Age would be challenging to interpret because of its endogeneity with these variables (i.e., 20-year-old female student is less likely to be currently pregnant vs. a 30-year-old male student's wife). If the survey had included age in categories sufficiently narrow to evaluate policy implications, respondents would have been uniquely decided (i.e., respondents' anonymity would not have been protected).

## Conclusions

Further analysis of a previously reported survey of University of South Dakota medical students shows items to be included in a policy on pregnancy and parental leave (Table 1). The responses did not differ significantly among demographic groups. Results show that knowing these items is useful based on many fewer than half of surveyed U.S. medical schools, including all seven items. Adding these items to public websites is an easily actionable intervention for medical schools to help current and future students who are parents or are considering parenthood.

## Availability of Data and Material

https://doi.org/10.25820/data.006159.

## Code Availability

https://doi.org/10.25820/data.006159.

## Ethics Approval

The University of Iowa determined that the collection of data from publicly available websites does not meet the regulatory definition of human subjects research.

## Supplementary Material

Supplemental data

## Notes

^*^
One exception was that one medical school uses two websites, the second referred to from the first, but blocked from Google search. A policy PDF created November 9, 2020 was downloaded from the second public website on April 18, 2021 and added to the school's PDF file (see Discussion).

## References

[1] Association of American Medical Colleges. Medical School Graduation Questionnaire, 2020 All Schools Summary Report, 2020. Available at: https://www.aamc.org/system/files/2019-08/2019-gq-all-schools-summary-report.pdf Accessed April 12, 2021.

[2] Kraus MB, Talbott JMV, Melikian R, et al. Current parental leave policies for medical students at US medical schools: A comparative study. Acad Med 2021;96:1315–1318. 33769337 10.1097/ACM.0000000000004074

[3] Durfey S, White J, Adashi E. Pregnancy and parenting in medical school: Highlighting the need for data and support. Acad Med 2021;96:1259–1262. 33570853 10.1097/ACM.0000000000003988

[4] Bye EM, Brisk BW, Reuter SD, Hansen KA, Nettleman MD. Pregnancy and parenthood during medical school. South Dakota Med 2017;70:12. 29334444

[5] Araújo J, Bacelar S, Jesus LE. Family planning among female medical students: Are their plans comparable to other professionals? Rev Assoc Méd Bras 2020;66:485–490. 32578783 10.1590/1806-9282.66.4.485

[6] Kuder-Richardson formulas. (2021). Available at: https://en.wikipedia.org/wiki/Kuder-Richardson_formulas Accessed April 17, 2021.

[7] McCracken CE, Looney SW. On finding the upper confidence limits for a binomial proportion when zero successes are observed. J Biom Biostat 2017;8:338.

[8] US News & World Report. 2021 Best Medical Schools: Primary Care, 2021. Available at: https://www.usnews.com/best-graduate-schools/top-medical-schools/primary-care-rankings Accessed January 18, 2021.

[9] Riano NS, Linos E, Accurso EC, et al. Paid family and childbearing leave policies at top US medical schools. JAMA 2018;319:611–614. 29450516 10.1001/jama.2017.19519 PMC5838606

[10] Magudia K, Bick A, Cohen J, et al. Childbearing and family leave policies for resident physicians at top training institutions. JAMA 2018;320:2372–2374. 30535210 10.1001/jama.2018.14414 PMC6583066

[11] Vance MC, Riano NS, Jagsi R, et al. Assessment of paid childbearing and family leave policies for administrative staff at top US medical schools. JAMA Int Med 2020;180:589–592. 10.1001/jamainternmed.2019.6653 PMC6990862 31961376

[12] University of South Dakota Sanford School of Medicine Campus Locations, 2021. Available at: https://www.usd.edu/medicine/our-locations Accessed March 26, 2021.

[13] Sterling HM, Allan BA. Construction and validation of the quality of maternity leave scales (QMLS). J Career Assess 2020;28:337–359.

[14] Suresh S, Hoffman R, Liu S, Gosbell A. Australian medical student expectations of work-life balance as a doctor. MedEdPublish 2020;9:256. 10.15694/mep.2020.000256.1 PMC10697482 38058950

