# Supplementary material for: Elements of Pregnancy and Parenthood Policies of Importance to Medical Students and Included in a Sample of Medical Schools' Websites and Student Handbooks
Source: Womens Health Rep (New Rochelle). 2021 Nov 29;2(1):533–41. doi: 10.1089/whr.2021.0105 (PMC8665277; doi:10.1089/whr.2021.0105)
Supplement: Supplemental data [file Supp_Data.pdf]

This document is scanned copy of the screenshots sent to us:

Bye EM, Brisk BW, Reuter SD, Hansen KA, Nettleman MD. Pregnancy and parenthood during medical school. South Dakota Med 2017;70:12. PMID: 29334444

At https://FDshort.com/UnivSD, there are also:

33 PDF files one for each of the N = 33 surveyed schools' websites and manuals

Excel worksheet of findings of the survey of the N = 33 schools

Copy of website from US News & World Reports showing the N = 33 schools

University of Iowa's IRB approval

Statistical analyses showing the 245 associations calculated

UNIVERSITY OF
SOUTH DAKOTA
SANFORD SCHOOL OF MEDICINE

Pregnancy and Parenthood In Medical School Survey

Thank you for agreeing to participate in our research by completing the survey. We are striving to improve the way our medical school supports students who are pregnant or who are parents. All responses are confidential and you will not be asked to provide identifying information.

---

1) Are you:

○ Male [Value=1] ○ Female [Value=2] ○ Other or prefer not to answer [Value=3]

2) How many children do you have?

☐

3) Are you planning to have additional children during medical school?

○ Yes [Value=1] ○ No [Value=2]

4) How many children did you have prior to coming to medical school?

☐

5) Are you (or your significant other) currently pregnant?

○ Yes [Value=1] ○ No [Value=2]

6) Were you (or your significant other) pregnant when you started medical school?

○ Yes [Value=1] ○ No [Value=2]

7) If you have a significant other, is he/she also a medical student?

○ Yes [Value=1] ○ No [Value=2] ○ No current significant other [Value=3]

8) Does your medical school have a written policy/document addressing pregnancy/maternity leave during medical school?

○ Yes [Value=1] ○ No [Value=2] ○ Unsure [Value=3]

9) What do you think are the key elements that should be included in a policy on pregnancy/maternity leave? Check all that apply:

☐ The school's plan for communicating the policy to the students [Checked=1]
☐ A statement indicating that the medical school wishes to be supportive of pregnancy and parenthood in medical school [Checked=1]
☐ How a student arranges for maternity/paternity leave [Checked=1]
☐ How the student and school jointly devise a plan to make up required elements that are missed during leave or because of pregnancy [Checked=1]
☐ How the student receives/requests additional accommodations/flexibility if pregnancy or breastfeeding interferes with expected activities [Checked=1]

☐ How much time a student can take off during medical school and still graduate with their class [Checked=1]
☐ Resources available for students who desire mental health counseling [Checked=1]
☐ Other (please specify) [Checked=1]

10) What are the most important things that a medical school could do to support a female medical student during her pregnancy and post-partum period (please list at least one)?

(1000 characters remaining)

11) What are the most important things that a medical school could do to support a male medical student during his significant other's pregnancy and post-partum period?

(1000 characters remaining)

12) What are the most important things that a medical school could do to support medical students who are parents (please list at least one)?

(1000 characters remaining)

13) Are you a parent or are you (or your significant other) currently pregnant?

○ Yes [Value=1]
○ No (If no, survey ends here. Please scroll to the bottom of the survey and select the Continue button to submit your survey) [Value=2]

14) Do you receive any help from the medical school in terms of being a parent?

○ Yes [Value=1]
○ No [Value=2]

15) If yes, what type of help?

(1000 characters remaining)

16) What are your feelings with regard to this help?

○ Extremely satisfied [Value=1]
○ Satisfied [Value=2]
○ Neutral [Value=3]
○ Dissatisfied [Value=4]
○ Extremely dissatisfied [Value=5]

17) If you currently have children, what do you utilize for routine daily childcare? Check all that apply:

☐ Significant other [Checked=1]
☐ Daycare [Checked=1]
☐ Other family member (mother, aunt, etc) [Checked=1]

- ☐ Friend [Checked=1]
- ☐ Nanny [Checked=1]
- ☐ Preschool/Montessori [Checked=1]
- ☐ I do not currently have children [Checked=1]
- ☐ Other (please specify) [Checked=1]

18) What are the major challenges of pregnancy/parenthood that you have faced during medical school training (please list at least one)?

(1000 characters remaining)

19) Were/are you or your significant other pregnant at any time during medical school?

- ○ Yes [Value=1]
- ○ No (If no, survey ends here. Please scroll to the bottom of the survey and select the Continue button to submit your survey) [Value=2]

20) Did you receive any support from the medical school in terms of pregnancy or the postpartum period?

- ○ Yes [Value=1]
- ○ No [Value=2]

21) If yes, what type of support did you receive?

(1000 characters remaining)

22) What are your feelings with regard to this support?

- ○ Extremely satisfied [Value=1]
- ○ Satisfied [Value=2]
- ○ Neutral [Value=3]
- ○ Dissatisfied [Value=4]
- ○ Extremely dissatisfied [Value=5]

23) What complications have/did you (or your significant other) encounter in pregnancy during medical school? Check all that apply:

- ☐ Pregnancy-Induced Hypertension [Checked=1]
- ☐ Pre-eclampsia [Checked=1]
- ☐ Eclampsia [Checked=1]
- ☐ Preterm labor [Checked=1]
- ☐ Premature rupture of membranes [Checked=1]
- ☐ Low Birthweight [Checked=1]
- ☐ Postdates [Checked=1]
- ☐ Hyperemesis [Checked=1]
- ☐ Preterm delivery [Checked=1]
- ☐ Bleeding [Checked=1]
- ☐ Miscarriage [Checked=1]
- ☐ Stillbirth [Checked=1]
- ☐ None of the above [Checked=1]
- ☐ Other (please specify) [Checked=1]

24) If you had a complication with your pregnancy or labor and delivery, did your provider require you to take time off during pregnancy or the post-partum period?

○ Yes [Value=1]
○ No [Value=2]

25) If yes, what was the provider-required length of time?

○ No time off [Value=1]
○ < 1 week [Value=2]
○ 1-2 weeks [Value=3]
○ 3-4 weeks [Value=4]
○ 5-6 weeks [Value=5]
○ > 6 weeks [Value=6]

26) How much time did you take off during the pregnancy, which was not required by a provider?

○ No time off [Value=1]
○ < 1 week [Value=2]
○ 1-2 weeks [Value=3]
○ 3-4 weeks [Value=4]
○ 5-6 weeks [Value=5]
○ > 6 weeks [Value=6]

27) What type of delivery did you have?

○ Vaginal [Value=1]
○ Cesarean [Value=2]

28) Did your baby (ies) have any complications in the neonatal period?

○ Yes [Value=1]
○ No [Value=2]

29) If yes, please describe

(1000 characters remaining)

30) Was your baby (ies) admitted to the Neonatal Intensive Care Unit?

○ Yes [Value=1]
○ No [Value=2]

31) How much time did you take off for maternity/paternity leave after delivery?

○ No time off [Value=1]
○ < 1 week [Value=2]
○ 1-2 weeks [Value=3]
○ 3-4 weeks [Value=4]
○ 5-6 weeks [Value=5]
○ > 6 weeks [Value=6]

32) In retrospect, would you have taken more time off for maternity or paternity leave if: (Check all that apply)

☐ Additional time off did not delay graduation? [Checked=1]
☐ Additional time off reduced the number of elective rotations you could take but did not delay graduation? [Checked=1]
☐ Additional time off delayed graduation by 1 month? [Checked=1]

33) What other factors might have allowed you to take more time off?

(1000 characters remaining)

34) Has pregnancy/parenthood affected your decisions with regard to the following (Check all that apply):

☐ Specialty choice [Checked=1]
☐ Interviews for residency [Checked=1]
☐ Choice of electives [Checked=1]
☐ Away rotations [Checked=1]
☐ Campus selection [Checked=1]
☐ Other (please specify) [Checked=1]

Other:

35) What insurance product did/do you use for your or your significant other's pregnancy?

- ○ Private insurance [Value=1]
- ○ Medicaid [Value=2]
- ○ Other (please specify) [Value=3]

36) If you have other suggestions about how a medical school could help support students who are pregnant or who are parents, please list them below:

(28000 characters remaining)
